# Biomechanical signaling of cytoskeleton and membrane reorganization in tumor immune chemotactic migration

**DOI:** 10.7150/thno.134995

**Published:** 2026-06-04

**Authors:** Qijie Zhao, Keliang Chen, Minglu Zhou, Jing Jin

**Affiliations:** 1 Department of Pharmacy, West China Hospital, Sichuan University, Chengdu, 610041, People’s Republic of China.; 2 Department of Biotherapy, West China Hospital, Sichuan University, Chengdu, 610041, People’s Republic of China.; 3 Department of Radiation Oncology, Cancer center, West China Second University Hospital of Sichuan University, Chengdu, People’s Republic of China.; 4 Key Laboratory of Birth Defects and Related Diseases of Women and Children (Sichuan University), Ministry of Education, Chengdu, People’s Republic of China.

**Keywords:** Chemokine receptors (CKRs), Chemotaxis, Actin, Cytoskeleton, Metabolic reprograming

## Abstract

A prominent activity of the chemokine system is the regulation of immune cells trafficking in tumor microenvironment (TME). This elegant chemotaxis process is regulated by both chemokine receptors (CKRs) stimulation and intracellular factors, such as chemokine, actin cytoskeleton, cell adhesion molecules, membrane fluidity, metabolic reorganization that govern differences in tumor immune cells migration patterns. At the core of tumor immune cell migration and effector functions are three fundamental questions: how CKR signaling triggers morphological changes, how the interplay between actin/selectin and energy metabolism regulates force production, and how cytoskeletal signaling adapts to membrane fluidity. In this review, we discuss the potential influences of CKRs signaling of the actin/selectin cytoskeleton reorganization, membrane fluidity, force regulation, and metabolic reorganization in immune cell chemotaxis. Apparently, the activity of immune cells migration is not regulated solely by chemokine-receptors recognition, as biomechanical-metabolic signaling orchestrate is critical for migratory morphology regulation. By using current knowledge of general chemotaxis mechanisms as a framework, we hope to provide new insights into secondary changes mechanisms of cytoskeleton and morphology network in supporting the tumor immune cells migration. Unraveling the mechanisms underlying chemotaxis and coupled mechanical immune checkpoints holds immense potential for discovering novel therapeutic targets in cancer immunotherapy.

## Introduction

The chemokine relevant immune contexture is widely recognized as an important determinant of outcomes in cancer patients^1^. Chemokine constitutes a large family of signaling proteins that interact with cell surface chemokine G protein-coupled receptors (GPCRs) 2. The cross-talks between chemokine and their receptors (CKRs) regulate immune cells activation and trafficking in physiological and pathological conditions^3^. Tumor microenvironment (TME) is associated with chemokine signaling induced intricate intracellular network and metabolic changes, especially tumor and immune cells^4^. Specifically, among the TME, accumulating evidence shows that tumor-derived chemokine imposed several limitations to dampen T cell immunity, and immunosuppressive cells experienced hyperactivation^5^. Although the chemokine determined chemotaxis are crucial for migration of distinct type of immune cells^6^, the physiological correlation between intracellular kinesin and structural proteins in immune cells remain unclear.

The immune cytoskeleton is composed of dynamic networks of filaments that drive cell shape, polarity, migration, and the assembly of intercellular contacts 7. During the immune cells chemotactic migration, CKRs, integrins and the cytoskeleton are triggered in a hierarchical sequence^8^. Parameters of TME immune cell migration are deeply influenced by CKRs signaling, and alterations in cells speed and directional persistence were result in a flawed location strategy and defects in immune priming^6^. Precise spatiotemporal control of these processes is essential to launch an effective innate immune response^9^, ^10^. The chemokine-induced CKRs stimulation is coupled to G-protein and β-arrestin-mediated signaling cascades, which in turn, are coupled to ultrastructural changes and molecular processes that define immune cells phenotype and function^11^. Morphological fitness of immune cells, defined here as their propensity to remodel cell shape in response to motility and chemokine stimuli, therefore affects their efficiency in the TME^12^. Insight into the role of CKRs signaling in the direct extravasation of immune and immunosuppressive cells is important from the angle of molecules composing the complex cytoskeleton-reorganization machinery^13^, ^14^. While some intricate details of the immune cell dynamics mechanisms in TME remain unknown, there have amount knowledge available to aid in the progression of our understanding.

Another key facet of chemotaxis control relates to the high energetic cost of actin polymerization^9^. As immune cells like Tregs experience the metabolic framework of growing tumors, they activate distinct pathways necessary to accomplish their function^15^. Sustained chemokine-induced adaptations in immune cells require metabolic activity and endogenous signaling cascades, with changes in glycolysis and cholesterol metabolism emerging as prominent features^16^, ^17^. However, several lines of evidence indicate that the underlying situation is more complex. Understanding the immune cells chemotaxis relevant CKRs signaling and metabolic switch in the TME is crucial for improving immune cell-based therapies.

In this review, we highlight current knowledge of CKRs signaling alter the immune cells migration and organellar proteins possessing in the context of tumor, as well as signal transduction, cells polarization and metabolic adaptation. We further examine how these studies illuminate the ways in which CKR signaling–induced conformational changes enhance the regulation of intracellular molecules within the structural network of leading edge and the trailing edge (uropod), thereby improving morphological fitness and influencing immune cell migration and functions.

## Chemokine and receptor recognition

**Figure 1:**
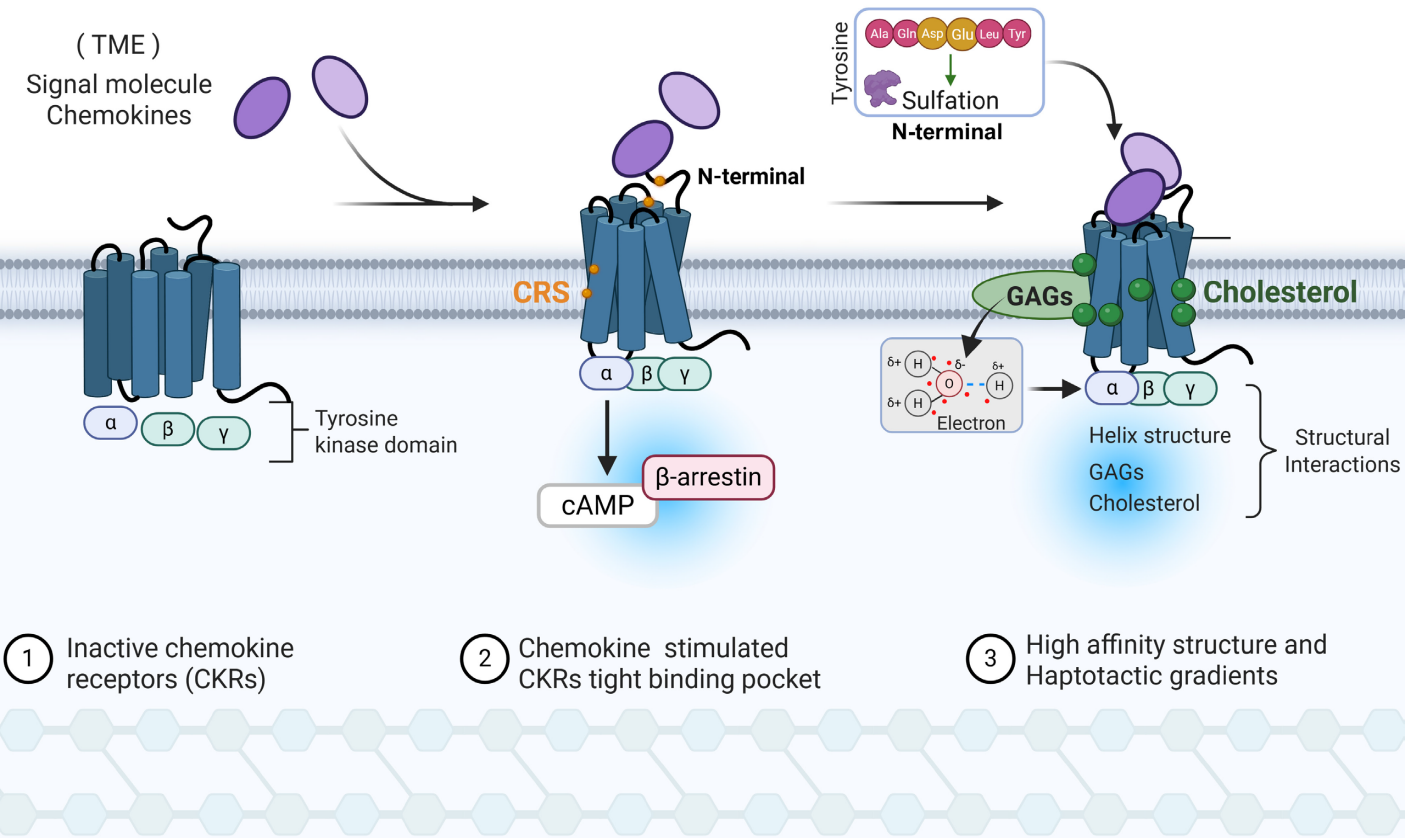
** Examples of immune CKRs activation structural model**. This figure illustrates the structural and molecular recognition mechanisms of CKRs in TME immune cells. **I.** CKRs recognition and binding: The chemokine's N-terminus length and conformation dictate its insertion degrees in the CKR's transmembrane pocket, wherein the receptor's N-terminal domain dynamically interacted with chemokine by multiple sites. **II.** Stabilization and affinity reconstruction: Cellular cholesterol stabilizes activated-CKRs structural through hydrophobic interactions. Meanwhile, N-terminal tyrosine sulfation n CKRs could enhance the binding affinity by electrostatic interaction with GAGs. **III.** Immune cells activation and signaling transduction: After chemokine-CKRs recognition, the chemokine stimulated the helical structural rearrangement and forming a tight activation CKRs pocket. Additionally, G proteins and β-arrestin cooperation stimulates the structural rearrangement and downstream cAMP and kinase signaling cascades. cAMP: Cyclic adenosine monophosphate, CKRs: Chemokine receptors, GAGs: Glycosaminoglycans, CRS: N-terminal domain of the receptors, TME: Tumor Microenvironment.

CKRs, a family of small proteins (8–10 kDa) activated by chemokines, are crucial in driving immune microenvironment dysregulation and tumor progression^18^. Recognition between CKRs and chemokines exhibits diversity and promiscuity, involving multiple interaction interfaces that contribute to biased signaling and functional selectivity^19^. With the physical combination between chemokines and CKRs, cell exerts its structural and physiological functions change in TME (**Figure 1**).

Chemokines N-terminal residues length determined its ability to penetrate the CKRs transmembrane helices binding pocket, with longer residues having deeper interior surfaces binding regions and selectively contributing to signaling biased agonism^20^. In compared with CCL5, CCL7 composed by longer residue and is more inclined to stimulate cAMP than β-arrestin signaling, one reason is binding to receptor residues closer to the intracellular side of the CCR1 conformations^20^. Qin *et al*. observed that CKRs N-terminus presented with conformation perpendicular to the membrane and extracellular half of helix shifted outward to forma top extra α-helical bending when interacting with chemokines^21^. A similar phenomenon was existed in CXCR2 when binding with CXCL8^22^. Meanwhile, different chemokine conformation like β-sheet interactions (CC motif) and no substantial protein-protein interface contacts (CXC motif), determine the recognition and interaction of CKRs type. Currently, multiple chemokine-CKRs complexes dynamic interaction sites have been proposed, termed as CRS1, CRS1.5, CRS2, etc.^23^. The signaling amplitude depends on the extent to which the receptor N terminus and CRS bind the chemokine, where different chemokine N-loop and distal flexible N-terminus epitope recognition acting as important initiators of signaling^24^. In particularly, human class A GPCRs subgroup of CKRs (CCR6, CCR7, CCR9, and CCR10) contain specific amino acids residue position, thereby only allow cognate chemokines with shorter N termini featuring and shallow binding mode^25^. After CKRs and chemokine interactions, CKRs presented with a tight ligand-binding pocket compared with the chemokine-free CKRs due to the inward shift of helix and extracellular loop^26^.

In the chemokine and CKRs recognition, the CKRs extracellular N-terminal regions sulfated tyrosine residues increases chemokine binding affinity and potency in initial binding site 27. Negatively charged residues (Asp and Glu) in the vicinity of tyrosine residues and these constitute the sequence motifs necessary for tyrosine sulfation. As a native pre-recognition complex modulation, tyrosine sulfation allowed CKRs to have more open and dynamic conformational states and facilitate ligand binding, as well as maintaining the long-range effects for the post-recognition 28. As a feedback, tyrosine sulfation binding to CKRs-sulfopeptides can modulate the oligomerization state of chemokines and affect the ability of a chemokine to activate its receptor 27. On the other hand, chemokines (basic) binding sulfated glycosaminoglycans (GAGs, acidic) selectively increase its concentration in the cell membrane with electrostatic and hydrogen bonding forces, wherein dimeric form interaction presented with higher affinity to CKRs^29^. The diminished affinity of chemokines for GAGs containing different heparin, heparan sulfate (HS) and chondroitin sulfate (CS) fine structures will directly influence the chemotactic and haptotactic gradients during the CKRs recognition. In addition, the activated CKRs structure is flourished with cholesterol, which binds at a specific transmembrane surface cavity and forms hydrophobic interactions with nearby residues^30^. The accumulated cholesterol could modulate CKRs-chemokine binding properties through orthosteric- and/or allosterical action. For instance, due to site-specific cholesterol consensus motif, cholesterol stabilizes the unique active conformation and maintains the tight helix structure of CKRs^31^.

## Mechanisms of CKRs signaling induced adhesive migration and morphogenetic processes

**Figure 2:**
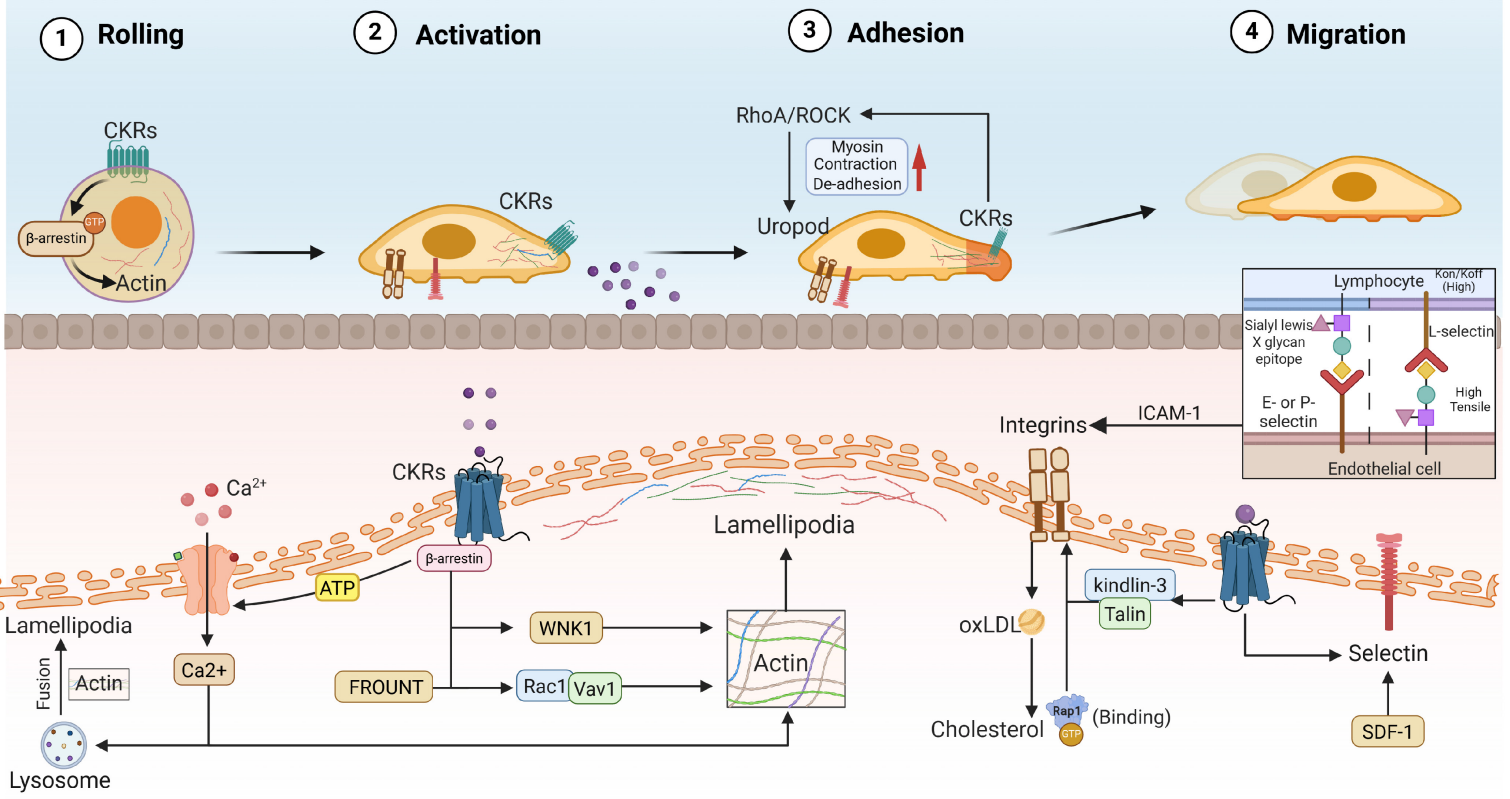
** Formation of branched actin networks and CKRs signaling in immune cells migration**. Immune cells morphological changes and polarization status modulated by transmembrane CKRs and cytoskeletal signaling in chemotaxis. **I.** Chemotactic rolling and adhesion: inside-out CKRs signaling like Talin and Kindlin-3 induced high-affinity conformations in integrins. Meanwhile, intercellular selectin recognition promoting the immune cell adhesion and rolling during the chemotaxis. **II.** Leading edge protrusion and actin polarization: Through the downstream β-arrestin, FROUNT, and Rac1/Vav1 signaling cascades, CKRs activation would promoting the actin polarization network and lamellipodia formation. **III.** Uropod contraction and de-adhesion: CKRs stimulates local intramembrane RhoA/ROCK signaling and non-muscle myosin rearrangement, wherein lysosome-modulated Ca²⁺ influx promote trailing edge contraction and detachment, thereby generating continuous forward force. CKRs: Chemokine receptors, oxLDL: Oxidized low-density lipoprotein, ICAM-1: Intercellular Adhesion Molecule 1.

Cell activation and migration are complex processes involving CKRs signaling, spatial allostery, internal mechanical and biochemical mechanisms that coordinately generate forces governing cell structure and directionality^32^. In recently, targeting the intracellular actin cytoskeleton and depolymerization by ultrasmall diameter magnetic nanomotor has been applied in improving tumor immunotherapy resistance and TME^33^. In classical theory, actin produces force for diverse cell motile and morphogenetic processes, wherein actin filaments act as ‘tracks’ for myosin motor proteins and move concentrated towards ATP energy on the intracellular membrane side 13. Actin-driven protrusions occur primarily at the front and myosin-driven contractile forces generate at the rear, causing cells to detach and move forward. Hence, intracellular force production and chemotaxis signaling are important for membrane deformation in many cellular processes (**Figure 2**).

### Actins

Due to the β-arrestins scaffolding capacity in the actin assembly events is needed for gradient-sensing filopodia and lamellipodia formation, elongation of actin filaments extend lamellipodia ends and push the plasma membrane to form protrusion, therefore β-arrestins activation is important for cytoskeletal reorganization and initiation of cell migration^13^, ^34^. Actin polymerization and assembly within cellular protrusions enhance outward forces^35^. However, to date, only β-arrestin_1 has been observed to be associated with CXCL12-induced actin polymerization and directed cells (DCs) migration^36^, which is closely associated with cognate CXCR4 nano-clustering and dynamics^37^. The Chemokine induced migration is a complex process involved in myriad proteins and intracellular pathways that coordinately to guide the polarized cell status and direction. Noteworthy, CKRs are usually concentrated at the leading front of motile cells, the membrane protrusion with correct receptor allows cell to positively sense chemoattractant gradients^38^. CXCL12 interaction promoted CXCR4 to form large nano-clusters with more than three receptors on the cell membrane, and this conformational intensification is important for receptor complex diffusion rates reduction, cell chemotactic sensitivity, actin cytoskeleton reorganization and integrin activation^38^. The CXCR4 receptor nanoclustering were temporarily confined in a region delimited by the F-actin cytoskeleton, which potentially increases the signal transmission stability. Omri *et al*. reported that actin polymerization drives actomyosin retrograde flow, generating “pushing” forces toward the membrane edge in immune cells, as well as contractile forces that “pull” the actins network to the leading front^39^. Additionally, upstream singling stimulation could accelerate actin dynamics and retrograde flow, consequently enhancing the actin polymerization and forces in immune cell migration^39^. In the case of T lymphocytes, pre-clustering of TCR oligomers with increased avidity has been reported to increase sensitivity to multivalent antigens by 100- to 3,000-fold^40^. Similarly, CCR5 and CCR7 transport to the plasma membrane also boosts the concept of CKRs local and polarized migration signaling^41^. The CCR7 activation triggered endomembranes Vav1/Rac1 signaling shows the ability to promote lead edge actin cytoskeleton reorganization and lamellipodia formation, subsequently promoting membrane protrusion and DCs migration^41^. Herein, CKRs activation induced local morphology change supporting the endomembrane actin signaling transduction and sustained directed migration. CCR7 signaling-induced ion influx kinase WNK1 was proved to be activated at the leading edge of T cells, thereby promoting osmosis- swelled the membrane at the leading edge and generating space where actin filaments can polymerize^12^. In addition, through binding with CCR2 or CCR5, intracellular protein FROUNT should amplify the chemokine-elicited Rac1-lamellipodium protrusion cascade^42^. FROUNT forms a complex with leading edge CCR2 microclusters and enhances the lamellipodium protrusion activity, which contribute to morphology asymmetric distribution and could be a mechanical immune checkpoint in immune disease. Hwang *et al*. demonstrated that exposure CXCL13 to CXCR5 would promote B cells three-dimensional lamellar-like pseudopods and F-actin-rich ridges in a short time^43^. On the other hand, CKRs-stimulated RhoA localizes to the cellular trailing edge, where RhoA/ROCK signaling promotes non-muscle myosin activation, uropod contraction and cell de-adhesion 44. Local activation of RhoA not merely promoted actin and myosin recruitment, but also increased intracellular stress fibres flow-forced traction forces and actin flow^45^. CKRs are embedded in lipid membrane, at least partially, and its nano-clustering and dynamics might be influenced by membrane fluidity and actin polarization.

### Ca^2+^

Chemokine activated CRKs have also be demonstrated to trigger Ca^2+^ flux and decrease cell adhesion, such as CXCR4, CCR5, etc. An increase in intracellular free Ca^2+^ is a sign of chemoattractant stimulation, and is linked to directional sensing, cytoskeleton structure, deformation tractive force and adhesions^45^, ^46^. Lorenzen *et al*. reported that activation of G protein subtype subunits through CCR5 leaded to Ca^2+^ flux enhancement, like G_q_, G_i_ and G_βγ_ protein activation, wherein G_βγ_ subunit release after CCR5-G_q_/G_i/o_ activation signaling bias potentially be responsible for eliciting Ca^2+^ flux^47^. With the CKRs concomitant ATP signaling, ATP releasing and degradation exerts a synergistic effect on Ca^2+^ flux through activating purinergic receptors and other G_i_ subunit^48^. As an important step in chemotaxis, Ca^2+^ flux is necessary for actin polymerization and cytoskeletal reorganization. Increased Ca^2+^ flux supports actin polymerization and ensures cells correct polarization and migration^49^. Due to the CKRs-induced Ca^2+^ flux, Ca^2+^-ATPases on the plasma membrane and endomembrane are responsible for Ca^2+^ homeostasis by pumping between extracellular space and intracellular stores^50^. Moreover, upon chemokine-stimulated Ca^2+^ flux, lysosomes fuse to the cell membrane in a localized area and contribute to local increases in ATP and lysosomal contents^48^. Especially, fusion of lysosomes with the plasma membrane at lamellipodia maintains cell shape remodeling and polarization through endomembranes replenishment and uropod disconnection^51^. Current research posits that lysosomes function as a signaling hub that integrates both extracellular and intracellular stimuli, thereby regulating cellular homeostasis^52^. Through post-fusion releasing with actomyosin cytoskeleton, lysosomes-Ca^2+^ signaling activated the actin-based motor myosin at the cell rear, ultimately facilitating the persistently intracellular force and immune cells migration^53^.

### Integrins

Chemokines-induced transmembrane adhesion protein (integrins) activation formed a critical link between the ECM and the cytoskeleton, as well as promoting the adhesion of immune cells to three-dimensional substratum^54^. As feedback, the activated integrins will drive excessive engagement and internalization of oxLDL, subsequently promoting the accumulation of cholesterol. Since chemokine-CKRs interaction caused a significant increase in the appearance of membrane actin-coupled protrusion (filopodia, lamellipodia and uropod), which are integrin-rich structures implicated in cell adhesion, and due to integrin-to-basement membrane for cells local/distal actin polymerization and forward movement^55^. Thus, in the transient cell-substrate interactions for movement, integrins and unspecific adhesion promoted forward force is highly dependent on actin polymerization and larger focal adhesions. Sheetz *et al*. has been indicated that integrins in the front of lamellipodia with enhanced cell cytoskeleton exhibit forward pull force to make itself moving 56. Through integrative tension sensor (ITS) technique, Ying *et al*. observed that filopodium elongation is accompanied with myosin and integrins enrichment during cell adhesion movement, wherein integrins tension could generate in discrete foci (force nodes) along single filopodia with a spacing of ∼1μm^57^. In addition, filopodia integrins tension shows region-dependent, with weak tensions generated in the nodes at the filopodia tips and strong tensions generated in the nodes at the filopodia bases^57^. Increased adhesion maybe attributed to the result of inside-out integrins activation in immune cells, which involves the activation of the Rap1-GTPase and the integrin adaptor proteins Talin^58^. Mechanistically, surface membrane integrins undergo conformational switching to a high-affinity state via chemokine binding activation. It is widely accepted that integrins such as LFA1 are kept in low-affinity states with bent/closed conformations at quiescent condition, extended/closed conformers (intermediate affinity) to mediate rolling, and open/extended conformers (high affinity) by CKRs signaling inside-out stimulation to promote arrest and firm adhesion^59^. Activation of CKRs induced a high-affinity integrin structure to the endothelial cell interaction, which is potentially required for the firm binding of immune cells adhesion migration. The integrins high affinity conformation and adhesion ability require kindlin-3/Talin-1 binding to the β2-integrin cytoplasmic domain under the chemokines-CKRs recognized activation^59^, ^60^. Although all integrins can undergo activation, β2 integrins are exclusively expressed on the surface of leukocytes, neutrophils, lymphocytes, and monocytes, and show different degrees of activation under ultimate talin-1 or kindlin-3 coupling signals 61. CKRs-activated integrins acted as a frictional interface with the environment, conveying tangential traction forces like a rake pulled.

### Selectin

In tumor, the functional selectins expression was proved to be important for immune cells homing^62^, ^63^. Selectins are single-chain transmembrane glycoproteins that resemble C-type lectins, found on the surface of immune cells and endothelial cells, especially within the vascular network. Selectins have rapid association and dissociation rate constant (Kon/Koff) and a high tensile strength, supporting the observed transient interactions 64. The selectin family which comprises 3 members like E-selectin, L-selectin and P-selectin bind to sialyl-Lewis x (sLeX) epitope carbohydrates^65^, wherein the vessel wall chemoattractant signals for lymphocyte is controlled by the diversity of the selectin. The chemokine-triggered immune cells orientation movement could be arrested by selectin blocking, such as CXCR2^+^ neutrophils in inflammation 66. Selectin- as well as chemokine-mediated inside-out signaling promoted efficient neutrophil recruitment 67. In terms of this, selectin-mediated CD11a/CD18 neutrophil activation initiates slow rolling by transient interactions with intercellular adhesion molecule-1 (ICAM-1), which allows neutrophil to sample chemokines presented by the endothelial cell wall and further activation of β2 integrins^68^. For CKRs stimulus like chemokine and SDF-1α, SDF-1α-specific upregulation of selectin in endothelial cells has been observed 69. Maryann *et al*. indicated that CXCR3-driven CD8^+^ T cells transfer is dependent on selectin and ICAM-1 activation, which is important for T cell trafficking across tumor vascular gateways 62. Thus, CKRs-induced selectin facilitated the immune cells rolling to firm arrest within the TME, supporting strategic intervention of chemokine-dependent delivery of immune cells for cancer treatment. The cooperation of CKRs and selectin not only facilitates the early tumor seeding, but also promotes the immune lymphocytes trans-endothelial migration^70^. For example, the selectin-positive tumor endothelial cells exhibited immune-related pathways enrichment and enhanced CD8^+^ T cells infiltration, wherein CCL5 synergistically induced ACKR1^+^CD8^+^ T cells recruitment and activation^71^. In gastric adenocarcinoma, increased levels of selectin and CCR4 in CD4^+^/Foxp3^+^ Tregs contribute to Tregs recruitment and tumor progression^72^. Targeting both chemokines and selectins improved the tumor progression and tumor immune microenvironment rather than inhibiting selecting 73. In rat sarcoma model, chemokine and selectin serum concentration were increased within tumor progress, whereas these parameters returned to baseline after tumor excision^74^. Collectively, investigating the cooperation among the CKRs signaling, actin cytoskeleton, cellular morphological changes and adhesion molecule, as well as underlying driving force, will provide insight into development of antitumor immunity dependent immune cell chemotaxis.

## CKR relevant immune cell metabolic reprograming

### OXPHS

**Figure 3:**
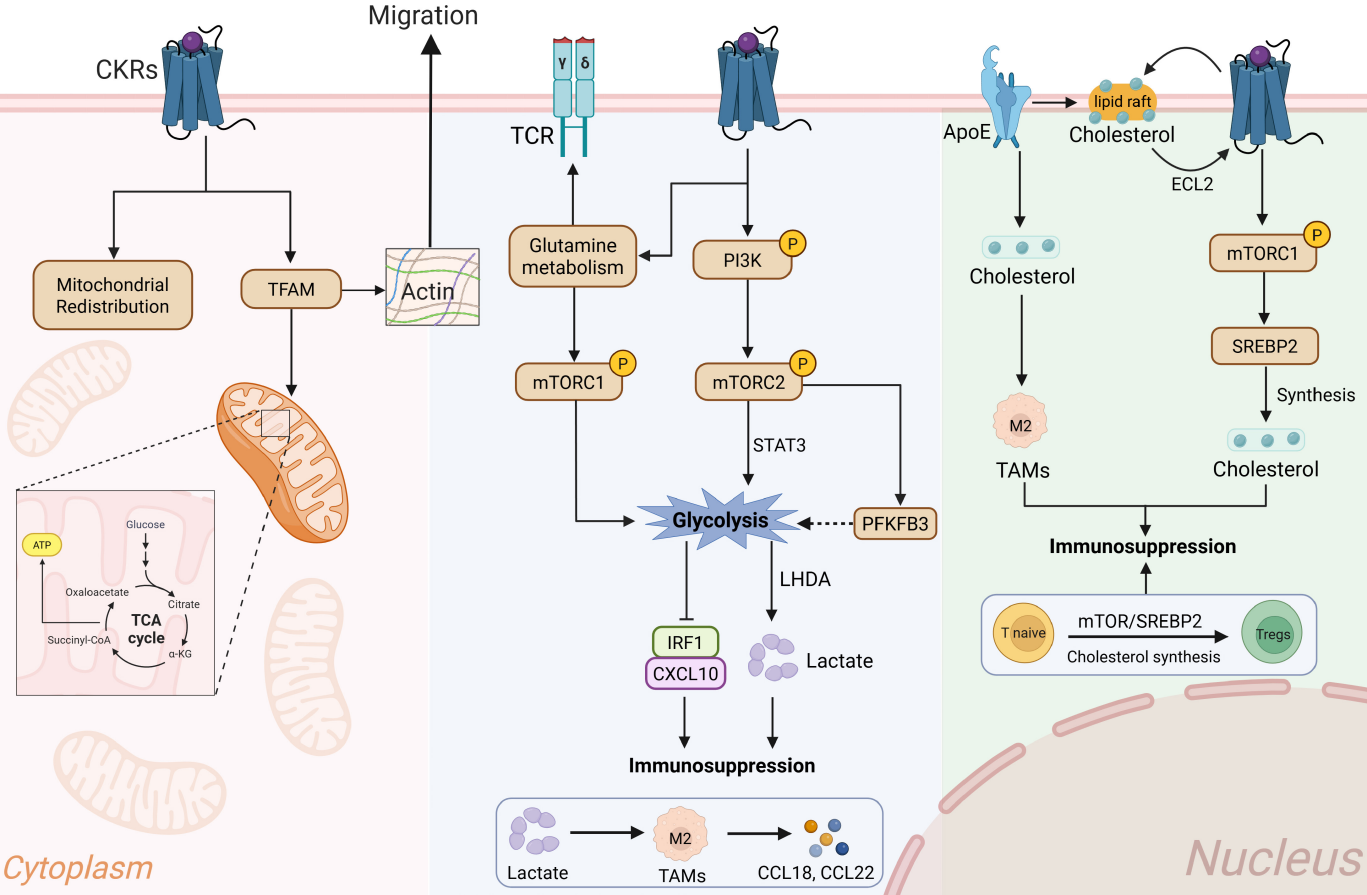
**Interplay between CKRs signaling and metabolic reprograming in immune cells**. The chemotaxis and cytoskeleton rearrangement are highly energy-dependent manners. Activated-CKRs signaling promote the intracellular metabolic reprograming and energy requirements. **I.** OXPHOS: CKRs activation promotes the TFAM synthesis and mitochondrial activity, wherein the enhanced TCA cycle and ATP generation maintain energy requirement for stable morphological activities. **II.** Glycolysis: In part immune cells, CKR-driven PI3K/mTOR and HIF-1 signaling pathways upregulated the glycolytic enzymes like LDHA and PFKFB3, subsequently achieving rapid local ATP for actin rearrangement and extension effects. In addition, lactate accumulation in TME promoting the immunosuppressive effects and M2-like TAM chemokine secretion. **III.** Lipid and cholesterol metabolism: ApoE-mediated lipid rafts involved in stability of CKR transmembrane conformation, as well as activating mTOR/SREBP2 cascade to promote membrane fluidity and actin polarization. Meanwhile, cholesterol synthesis and mTOR/SREBP2 signaling were coordinately regulating the Tregs and M2-like TAMs accumulation. TFAM: Mitochondrial transcription factor A, TCA: Tricarboxylic Acid Cycle, TCR: T Cell Receptor, OXPHS: Oxidative Phosphorylation, LDHA: Lactate Dehydrogenase A, TAMs: Tumor associated macrophages, CKRs: Chemokine receptors, Tregs: Regulatory T cells, ApoE: Apolipoprotein E.

In immune cells, processes such as cytoskeletal rearrangement, membrane trafficking, traction stress generation, migration, and proliferation all require a continuous energy supply. Mitochondria are usually located in the cytoplasm of cells, where they generate adenosine triphosphate (ATP) to empower cellular functions (**Figure 3**). Through real-time ATP biosensor and traction force microscopy, Curtis *et al*. observed positive correlation between cell traction forces (contractility) and energy consumption, indicating that enhanced cytoskeletal reorganization and integrins affinity require significant ATP utilization and glucose metabolic efficiency^75^. CXCL12-dependent leukocyte polarization and migration accompanied with mitochondrial redistribution^76^. Mitochondria respond to chemokine-stimulated and accumulated microtubule-organizing center. In gastric carcinomas, CCL21-CCR7 axis induced mitochondria pathway protects CD8^+^CCR7^+^ T lymphocytes from apoptosis^77^. The CKRs activation and mitochondrial metabolism energy supply in CD8^+^ T cells supported its high cytotoxicity and favor the interaction to tumor cells. In the cytoplasm, mitochondrial transcription factor A (TFAM) is essential for mitochondrial respiration and controls mitochondrial transcription, and packaging^78^. CKRs activation was proved to increase TFAM synthesis, thereby enhancing Th1 and Tregs responses^79^. The stable relationship between actin cytoskeleton and integrins adhesions may also result in a more efficient conversion of ATP to cellular traction forces, which is also critical for the regulation of intercellular adhesions and communications^75^. Due to TFAM is essential for mitochondrial respiration and ATP generation, TFAM-deficient Tregs presented with reduced proliferation and Foxp3 expression upon glucose deprivation *in vitro* TME^78^. Impaired mitochondrial ATP generation in Tregs contributes to an increase in CD8^+^ T cells, at least partially by reducing their glucose consumption and thereby alleviating glucose deprivation in the TME. Moreover, Eros *et al*. reported that decreased TFAM in immune cells will compromise the actin cytoskeleton and impair cellular motility in response to chemokine signaling, leading to spatial disorganization in the hypoxic TME^80^. The integrins induced P2X4 purinergic signaling and ATP generation is essential for effector immune cells adhesion and migration^81^. Li *et al*. reported that PEG-amine-QD nanoparticle exposure could stimulates purinergic signaling and early ATP release, driving integrin-dependent adhesion and crawling during immune cells recruitment^82^. Thus, targeting the ATP-dependent morphological dynamics interferes with the cellular interactions required for crawling and transmigration, thereby facilitating the clinical application of chemotaxis-targeted therapies.

In line with this, CXCR4 blocking significantly decreased mitochondria number and oxidative phosphorylation, and induced mitochondria loss related non-apoptotic T cell death in T cell acute lymphoblastic leukemia (T-ALL) 83. Mitochondrial inhibition potentially suppresses T cell activation through decreased myosin phosphorylation and ATP production, thereby weakening immune synapse adhesion strength and force transmission at the point of contact^84^, ^85^. Force-producing actin polymerization and actomyosin contraction as well as MMP delivery and membrane trafficking are energetically demanding processes 86. Of note, the anti-tumor chemokine CXCL14 could inhibit mTOR phosphorylation signaling and tumor cells proliferation 87. CXCL14-suppressed mTOR signaling and oxidative phosphorylation were also observed in leukemia-initiating stem cells and lymphocyte migration^88^. Targeting regulation mitochondrial respiration and metabolic reprograming may break the actin cytoskeletal reorganization dependent chemotaxis and warrants future consideration for certain cancer therapies.

### Glycolysis

Glycolytic metabolism is a hallmark of tumor immune microenvironment. In tumor cells, chemokine induced tumor glycolysis like glycolytic enzymes GLUT1, HK2, and LDHA play a crucial role in both tumor progression and metastasis^89^ (**Figure 3**). For tumor immunodepression, CCL20-CCR6 axis stimulated Tregs glycolysis and was deeply involved in its immunosuppressive effects^90^. The CCR6-induced Tregs activation closely associated with intracellular glycolysis, which simultaneously damaged the CD8^+^ T cells immune response and activity. The immune cells migration is thought to be primarily dependent on the glycolysis enzyme glucokinase, which is induced by the PI3K-mTORC2 signaling pathway. CCL20-CCR6 signaling induced the phosphorylation of Akt, mTOR, and STAT3 molecules in T cells and Tregs^91^. In compared with naïve T cells, the CKRs signaling more likely to enhance the glycolytic metabolism in Tregs. For Tregs, upregulation of glycolysis would promote actin-based cytoskeletal reorganization, thereby enhancing their migratory activity and immunosuppressive function^4^. The increased ATPase level and F-actin formation were significantly co-localized in Tregs. Similarly, CCR6 activation enhanced Tregs glycolysis and lactate production, and thereby achieving immunosuppressive activity toward CD8^+^ T cells^90^. Like other G protein–coupled receptors (GPCRs), CCR6 activation induces Tregs phospholipase C activation and release of intracellular Ca^2+^ levels 92. Of note, CCR6 was found to be more frequently present in Tregs than in CD8^+^ T cells, which might preferentially promote Treg glycolysis, enabling them to outcompete CD8^+^ T cells for extracellular glucose. The CCL20-CCR6 axis inhibition not merely decreased compensatory glutamine metabolism, but also promoted the efficacy of anti-PD-1 therapy^90^. Glutamine is intimately involved in maintaining redox homeostasis, and acts as a metabolic hub to increase immune cells mTORC1-mediated glycolysis and enhancing TCR signaling^93^. Fluorescence lifetime microscopy revealed that activated T cells exhibit transient glycolytic activity, which promotes actin remodeling associated with outward immune synapse and receptor formation 94. The enrichment of actin polymerization at the immunological synapse augments T cells outward forces^35^. The inhibitor of glycolysis, 2-deoxyglucose (2DG), leads to increased T cell activation potential in tumor^95^. Similarly, low levels of 2-DG would induce DCs metabolic stress and glycolytic reprogramming, subsequently enhancing CKRs expression and integrins-mediated immune cell adhesion^95^. In return, loss of adhesion force will lead to a significant downregulation of DCs intracellular metabolic rate and cellular ATP generation. During early stimulation, adhesion-dependent glycolytic activation in CKRS-stimulated DCs potentially enhances T cell activation, whereas glucose consumption appears to restrict DC responses and T cell activation at later time points. Intriguingly, in the depriving TME, higher lactate also facilitates M2-like polarization of tumor associated macrophages (TAMs) in a dose-dependent manner and exerts their tumor-supporting functions^96^. Under conditions of extracellular glucose deprivation, lactate-induced M2 macrophages exhibited an immunosuppressive and chemotactic profile 97. Cellular energy metabolism and actin-based cell dynamics are tightly coupled processes. TAMs synergistically induced by lactate and chemokines exhibit immunosuppressive effects associated with CD8^+^ T cell dysfunction and Tregs accumulation. Spatial analysis revealed that TAMs localize to tumor regions enriched for hypoxia-responsive signatures 97. Within these regions, the close physical proximity between TAMs and CD8^+^ T cells directly drives T cell dysfunction and exhaustion, though it remains incompletely understood whether metabolic competition plays a contributing driver. This phenotype was potentially fueled by glycolysis and characterized by actin cytoskeleton changes, cell protrusions, as well as adhesion and migration^98^, ^99^. To control these activities before and after polarization, macrophages continuously form actin-rich membrane protrusions and extend filopodia from their cell surface^99^. The lactate-derived TAMs had a significant higher mRNA level of CCL18, CCL22, and IL-10, which potentially forming a positive feedback loop^100^. Mechanistically, glycolytic reprogramming in macrophages could promote histone acetylation mediated chemokine transcription^101^. Intervention of 6-phosphofructo-2-kinase/fructose-2,6-biphosphatase 3 (PFKFB3) blocked an increased glycolytic flux under hypoxia, which is directly response to the PI3K/Akt/mTOR signaling cascade^102^. Inhibition of PFKFB3 reduced the cell-cell average junctional tension by perturbing glycolysis-driven actin dynamics and directly disrupting intercellular contact forces. Meanwhile, activated CXCR4/mTORC2 signaling axis contributes to downstream glycolysis regulator PFKFB3 and activates endothelial cells migration^103^, ^104^. During macrophages migration processes, ATP is rapidly consumed in the cytosol for the reorganization of cytoskeletal actin filaments and force expenditure^105^. Silencing CXCR4 can inhibit glycolytic metabolism, thereby directly influencing the macrophages polarization and migration^106^. The adaptive responses in glucose metabolism and glycolysis reprograming contribute to immunosuppressive cells actin cytoskeleton reorganization and chemotaxis activity.

In anti-tumor immunity, CXCL10-CXCR3 axis was observed to stimulate CD8^+^ T cells mTOR phosphorylation activation, thereby stimulating lactate dehydrogenase A (LDHA) mediated glycolysis and lactate generation^107^. Through F-actin polarization at the lamellipodia, chemokine-driven motility, and cellular adhesion, upregulated glycolysis promotes actin polymerization and density as an energy state integrator and sustains cell cytotoxic activity^108^. In cooperation with glycolysis, OXPHOS partially promoted F-actin content and cell elongation in the context of confined motility. The IRF1 and CXCL10 immunostimulatory molecules were usually reduced in highly glycolytic TME^109^. However, CXCR3-induced intracellular glycolysis promoted cells infiltration, ATP and tumor necrosis factor-α (TNF-α) secretion^107^. In this way, the CXCL10-CXCR3 and glycolytic genes additional signaling networks is important for CD8^+^ T cells survival, morphological fitness and cytotoxic activity in the surrounding glucose-deprived TME. In terms of this, raised fast ATP content and F/G-actin ratio indicated the CD8^+^ T cells immune synapses outward extension force and synaptic adhesion movement^108^. The mTOR metabolic signaling enhances glycolysis and plays a critical role in CD8^+^ memory T cells antitumor immunity^110^. Because memory T cells undergo extensive activation upon antigenic stimulation, they require a high glycolytic flux to sustain the bioenergetic demands and provide building blocks for cytoskeletal biomass^111^. In this process, the coordination between integrins and F-actin flow maintains high affinity within the immunological synapse, drives TCR clustering, and enhances ICAM-1 binding to facilitate the elongation of activated memory T cells^112^, ^113^. Of note, lactate-mediated inhibition of T cell motility was partially attributed to an interference with glycolysis activated upon engagement of the chemokine CXCR3-CXCL10 axis^9^. Chemotaxis of CD8^+^ T cells was suppressed by lactate and enhanced by glucose^114^, which may be attributed to decreased CXCL10 levels in a highly glycolytic TME. Mao *et al*. engineered a multifunctional nanoparticle that targets the tumor mechanical microenvironment and AMP signaling, thereby reducing lactate levels by 50%, promoting extracellular matrix (ECM) and cytoskeletal reorganization, and enhancing effector T cell infiltration^115^. Glycolysis increases immune cells cytokine expression, and TCR or CKRs stimulated immune cells glycolysis was essential for their activation, biomechanics shape, membrane structure alteration and adhesive migration^116^-^118^.

### Cholesterol

Chemokine-dependent immune cell recruitment requires cholesterol, and higher lipid and cholesterol enhanced macrophages infiltration in TME^31^, ^119^, ^120^ (**Figure 3**). It has been well known that cholesterol is an allosteric modulator of GPCRs and is important for active conformation of CKRs^31^. Cholesterol homeostasis is crucial for maintaining the deformability and fluidity of cell membranes, as well as the allosteric regulation of surface receptors. In the GPCR extracellular loop 2 (ECL2), cholesterol could induce site-specific conformational restraint and regulate CKRs signal transduction 121. Immune cells chemotactic activity requires cytoskeleton-dependent membrane dynamics, wherein insoluble cholesterol sustain the transmembrane receptor protein and actin cytoskeleton, especially actin bundles formation, immune synapse, F-actin dynamic polarization and cell adhesion migration^122^. For example, successful CXCR4-mediated signaling and migration required the cholesterol-rich membrane microdomains known as lipid rafts^123^. Activated T cells presented with the movement of two-dimensional surface lipid rafts along actin filaments^124^. The CXCR4 lipid raft association was closely associated with rich cholesterol level^125^. In this instance, the diverse assembly states of CKR complexes may mediate the formation of specific raft domains and their colocalization with actin polymerization at the immune synapse. In addition, higher cholesterol and lipid rafts levels were accompanied with CKRs increasing in immune cells, rather than glycolysis or other canonical metabolic pathways^126^. Lipid raft integrity and cholesterol microdomains are critical for stabilizing T cells CKRs signaling and promoting F-actin polymerization at the periphery of the immune synapse^122^. Of note, the accumulation of actin polymerization at the immunological synapse augments immune cells adhesion force, thereby enhancing cytotoxicity against cancer cells^35^. In tumor cells, CXCL18 induced mTORC1 signaling was proved to increase *de novo* cholesterol synthesis by activating sterol regulatory element-binding protein-2 (SREBP2)^127^. Targeting mTORC1/SREBP2 directly enhanced metabolic-immunosuppressive role in tumor 128, which implied that CKRs induced mTOR signaling might plays an important role in cholesterol and lipid rafts regulation in Tregs and macrophage^101^. Of note, upregulated SREBP2 expression and the accompanying elevation in cholesterol promote enhanced Ca^2+^ signaling and coordinated F-actin polymerization in localized immune cells, thereby contributing to immune cells migratory force^129^. Intriguingly, through boost cholesterol synthesis, mTOR/SREBP2 signaling can induce metabolic reprogramming of naive CD4^+^ T cells and promote their conversion into Tregs and immunosuppressive function^130^. The cholesterol-dependent membrane lipid rafts not merely promote the actin cytoskeleton interactions and cell morpho-mechanical alterations, but also influence the differentiation status of immune cells^131^, ^132^. For the membrane lipid rafts regions cholesterol transport, apolipoprotein E (ApoE) was a necessary metabolic modulator in various tumor related immune cells, including macrophage and T cells^133^. The ApoE expression modulated the levels and distribution of cholesterol, directly involved in actin-dependent cells elongation and migration morphology^134^, ^135^. The ApoE inhibition directly decreased M2-like TAMs proportions and migration, which would be further promoted by CCR5 knockdown^136^. Moreover, lacking of CCR5 signaling also presented with defects in ApoE expressed immune cells trafficking^137^. Together, metabolic reprogramming and enhanced cytoskeletal reorganization play an integral role in signal transduction and force generation for immune cell chemotaxis and expansion within the TME.

## CKRs signaling reshapes tumor immune cells metabolic-cytoskeleton coupling

### T cell

**Figure 4:**
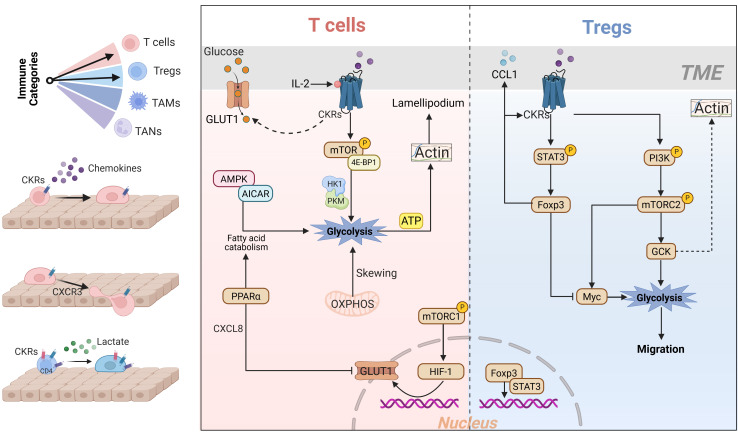
** Applying a mechanical load to T cells and Tregs chemotaxis requires actin network and metabolic reprograming cooperation.** CKRs-induced glycolytic reprogramming provides crucial biomechanical and energetic support for specific T cells chemotaxis, morphological adaptation, and effector functions. **I.** Effector T Cells biomechanical-metabolic signaling: CKRs-activated PI3K/mTOR, PPARα and AMPK signaling cascades in T cells shifts metabolism from OXPHOS and fatty acid catabolism toward glycolysis, thereby promoting rapid ATP generation. The CKRs-dependent rapid ATP generation enhance the T cells lamellipodial actin polymerization and physical penetration within tumor. Meanwhile, upregulated GLUT1 and glycolytic enzymes HK1/PKM were synergized with this effect. **II.** Tregs immunosuppressive metabolic reprogramming: Tumor-driven chemokines would promote CKRs-PI3K/MTORC2 based high glycolytic level and support Tregs chemotaxis and actin polymerization. Meanwhile, CKRs-stimulated STAT3/Foxp3 cascade could reshapes Tregs glycolytic metabolism to adapt depriving TME, thereby promoting immune synapse formation and enhancing migration and immunosuppression. GCK: Glucokinase, OXPHS: Oxidative Phosphorylation, Tregs: Regulatory T cells, GLUT1: Glucose transporter 1, Foxp3: Forkhead box protein P3.

Chemokine induced intracellular metabolic stress is associated with T lymphocyte migration and effect in the TME (**Figure 4**). The activated T cell adhesion and migration force were facilitated by ATP supplement and immune synapse-related actin cytoskeleton reorganization^81^, ^84^. The chemokine interaction with cognate CKRs simultaneously stimulates processes that increase intracellular nutrient and energy levels, thereby ensuring cell proliferation and biosynthetic processes. Kamnev *et al*. reported that the morphological fitness of T lymphocytes scales with the transcriptional activation of glycolysis, actin isoforms and membrane complex subunits^108^. In line with this, integrins induced effector T cells cytoskeletal reorganization and adhesion movement is ATP-dependent behavior^81^. Integrins activation signaling not merely increase the ATP generation, but also promote the Ca^2+^ to directly influence the membrane permeability. Both CD4^+^ and CD8^+^ T cells presented with increased cell size within 24 hours of activation, adapt the higher amounts of metabolites, and then followed by rapid cell division^138^. Meanwhile, T cell membrane domains and the actin cytoskeleton reorganized across increasing spatial scales, from highly ordered lipid rafts to filopodia and immunological synapses 139. In T cells, CCL5-induced GLUT-1 activation is important in glucose transport and facilitates glucose uptake, which is essential for actin polymerization and directional cell migration^140^. Moreover, CCL5-induced CCR5 signaling activates the mTOR/4E-BP1 pathway to directly modulate mRNA translation, nutrient sensing and glycolysis^140^, wherein activated mTORC1 in HIF-1/GLUT1 signaling is required for CD8^+^ T cells glucose metabolism and glycolysis in PI3K-Akt-independent manner^141^. Importantly, small changes in metabolic parameters are sufficient to lead to significant physiological cell migration changes, especially glycolysis-based rapid ATP production. Intervention of this process directly impaired the ATP generation and T cells migration, which was partially attributable to impaired adhesion and force transmission^94^, ^95^, ^140^. Mechanical forces spread across the T cells plasma membrane via a range of systems including integrins and adhesion proteins in conjunction with the actin cytoskeleton and mechanosensitive ion channels 139. Interestingly, although vertebrate glucose transporters belong to the GLUT family, GLUT1 does not appear to be expressed on quiescent T lymphocytes 142. With the membrane CXCR4 accumulation, GLUT1 expression was associated with increased glucose metabolism and higher migration ratio in immunopathy^143^. CXCR4 determined the fate of CD8-T lymphocytes immunological infiltration in type 1 diabetes^144^. In tumor, indigenous generation of particular chemokines force T cell activation and move towards the TME^145^. Lettau *et al*. reported that CXCR4-mediated HS1 phosphorylation is directly involved actin polymerization and adhesive migration, contributing to elongated cellular morphology and immunological synapse in T cells^146^. The TCR signaling transduction at the leading edge of T cells immunological synapse plays a crucial role in CXCR4-based chemotaxis^147^. The CXCR4 and F-actin were colocalized at the peripheral supramolecular activation cluster and redistribute by membrane kinetics in the T cells immune synapse^148^. It seems to support the concept that delivers adhesion receptors and proteins from the rear of the cell to the leading edge of the chemotaxis cell for extension and provides mechanical force to propel the cell forward.

Following activation, effector CD8^+^ T cells metabolic skews from infantile mitochondrial oxidative phosphorylation (OXPHOS) to glycolysis^138^, and is the major source of ATP to drive actin polymerization at the lamellipodium and related sustainment of motility^108^. Due to IL-2 ability in increasing CXCR3 and the migration of T cells in response to CXCL9 and CXCL10^149^, IL-2 induced CD8^+^ T cells glycolysis fitted the proposed notion of CKRs-aligned ATP production and actin cytoskeleton re-organization^108^. During early stimulation, CD8^+^ T cells undergo drastic morphological changes, with the formation of lamellipodia and immune synapses driven by a rapid ATP supply. Surprisingly, CXCR3 seems to be prevailing modulator in CD8^+^ T cells trafficking across tumor vessels, while CCR5 and CCR2 did not contribute this chemotactic response^150^. At the membrane terminus, CXCR3 stimulation triggered the effectors of early integrin-dependent adhesion, actin polymerization and motility^151^. Chow *et al*. reported that CXCR3 signaling is essential for the anti-PD1-mediated CD8^+^ T cells residing within tumors^5^. The glycolysis fueled the ability of effector T cells lamellipodium thickness, adhesion and orientation during confined chemotaxis, as well as their cytotoxic activity to tumor^5^. Meanwhile, the increased glycolysis level would promote T cells actin-associated immunological synapse formation and outward adhesion force^35^, ^94^. Given the causal link between actin polarization and T cell glycolytic activity 94, F-actin polarization and its binding to glycolytic enzymes may at least partially explain the robust enhancement of cellular glycolysis during chemotactic morphological reshaping. Intriguingly, the glycolysis and mitochondrial OXPHOS might in different ways to influence T cells morphology and chemotaxis, actin branching at the lamellipodium extension required local glycolysis-derived ATP, while limited-OXPHOS might promote a separate actin pool involved in uropod contractility^108^, ^152^. In the tumor severe nutrient deprivation milieu, hypofunctional CKRs responses are characterized by a gradual decline metabolism and gradient chemotaxis in effector CD8^+^ T cells, thereby contributing to an immunosuppressive microenvironment and poor anti-tumor immune response^153^. In this regard, upon the CXCR3 engagement with CXCL10 within lactate environment, significant up-regulation of two rate-limiting enzymes like HK1 and pyruvate kinase M1/2 (PKM) have been observed in the CD4^+^ T cells glycolytic pathway, rather than CD8^+^ T cells^9^. Specifically, CXCR3 signaling increased the glycolytic metabolism was directly linked to actin cytoskeleton reorganization and migration force^9^, ^108^. Despite of this, in tumor or non-tumor systems, CXCR3 is important for localization of CD8^+^ T cells, and interfere with recombinant cognate chemokines contributes to CD8^+^ T cell infiltration and tumor regression^150^. The CXCR3^+^CD8^+^ T cells chemotaxis was presented with increased level of F-actin polymerization, which drives their high migratory velocity^154^. Noteworthy, orderly cytoskeleton assembly actin polymerization after effector CD8 T cells activation could promoted synaptic autocrine of CCL5 and cytotoxic granules exocytosis during the in mechanical migration within TME^155^. In such self-feedback loop, the synergistic regulation between actin and the T cell local synaptic membrane facilitates adhesion and homing to target cells, thereby enhancing chemotactic efficiency. Besides, CCL5 and its cognate CKRs decreased AMPK/AICAR axis mediated fatty acid catabolism extent in T cell chemotactic response and principally switch to glycolysis and migration 156. Nguyen *et al*. observed that the co-localization of receptor CCR5 and actin at both the leading and trailing edges of T cells moving morphology, as well as the enrichment of cholesterol^157^. Generation of ATP from fatty acid catabolism in T-cell mitochondria is evoked by the stimulation of AMPK signaling through membrane receptor, intracellular Ca^2+^ or decreased intracellular ATP energy stress^158^, ^159^. Plausibly, in prostate cancer milieu, tumor-derived IL-8 will overactivated PPARα in CD8^+^ T cells, thereby decreasing glucose utilization by downregulating GLUT1 and HK2^160^. The agitation of cellular PPARα could reduce the use of glucose and promote the fatty acids utilization for metabolic needs 161. Impeding of glycolysis and fast ATP generation could paralyze CD8^+^T cells and slack the cellular morphological adaptation^108^, ^160^. In this regard, due to the effector CD8^+^ T cells metabolic skewing from infantile mitochondrial OXPHOS to glycolysis^138^, underscored the tight coordination between metabolic and actin polarization programs to sustain the T cells morphological adaptation forces, cytotoxic effects and chemotactic activity^108^, ^112^.

### Tregs

Tregs are a unique subset of CD4^+^ T cells that overcoming the hypoxic, acidic, and nutrient-deficient and maintaining immunosuppressive stress in tumor^162^. The high expression of CKRs likely drives the homing of Tregs to chemokine-rich microenvironments, where they suppress tumor antigen presentation and effector T cell responses through cell-cell interactions^163^ (**Figure 4**). The Tregs immunological synapse formation and migratory interaction are accompanied by the dynamic reorganization of the actin cytoskeleton^4^, ^164^. Generally, Tregs maintain homeostatic frequencies of 5%–10% in blood and lymphoid tissue, while its accumulation exceeding 50% of total T cells in tumor^165^. How to balance the systemic Tregs depletion induced secondary autoimmunity remains the ‘Sword of Damocles’ for current Tregs-targeted anti-tumor immunotherapies. Intra-tumoral Tregs can be distinguished from peripheral Treg cells and other effector T cells by high expression of the CKRs like CXCR3, CCR4 and CCR8^163^. The CXCL10-CXCR4 mediates migration of Tregs and intracellular F-actin polymerization and Ca^2+^ influx, and underlies the basis of Tregs leading edge migration and immunosuppressive functions^166^. The ability to form a stable immune synapse is essential for the proper function of Tregs, TCR/CD3-driven F-actin reorganization would increase in a few minutes after Tregs stimulation^167^. The lack of actin mobility will impair the TCR signaling, as well as naïve CD4^+^ T cells differentiation into Tregs subsets^166^. With chemokine induction associated metabolic stress, unlike thymus natural Tregs, peripherally induced Tregs are more likely to selectively accumulate by chemotaxis and reforming metabolism to selectively buildup of in tumor mass^168^, ^169^. Intriguingly, through adhesion interactions at membrane surfaces and immune synapses, Tregs can transfer their ATP-derived cAMP into DCs, thereby diminishing DC immunogenicity 170. However, with the CD74_deletion-mediated downregulation of Foxp3 expression and the inhibition of actin cytoskeleton organization, Tregs cell size and immune synapse formation are significantly reduced, thereby contributing to tumor rejection^171^. The cooperative engagement of CKRs and CD74 has been demonstrated to stimulate Ca^2+^ mobilization and F-actin polymerization, consequently promoting immune cells chemotaxis^172^. The successful establishment of CCL1-CCR8 interaction promotes Tregs Foxp3^low^ to Foxp3^high^ phenotypic characteristic in STAT3 phosphorylation dependent manners, as feedback, activated Foxp3^high^ Tregs will further promote CCL1 secretion and epitopes CCR8 expression to achieve self-feeding mechanism^173^. The CCL1-induced Ca^2+^ flux and membrane autocrine loop potentiate the Tregs suppressive activities. Plausibly, as a master-regulator of Tregs, Fxop3 not merely a co-transcription factor for Tregs, and also modulates intracellular glucose metabolism 174. In lactate rich and glucose deprivation TME, Fxop3-upregulation could repress Myc to restrict glycolysis and shifted cellular metabolism to OXPHOS, as well as compensating NAD^+^ consumption^175^. The glucose depletion and lactate production by tumors create a Tregs-favoring microenvironment, which may subsequently drive an immunosuppressive morphology and chemotactic interactions. Driven by tumor-derived cytokines and intracellular metabolic fueling, Tregs undergo actin cytoskeleton reorganization and exhibit enhanced mechanical deformability, which promotes their chemotaxis toward immune cells^4^, ^90^, ^176^. In this way, tumor can impair cytotoxic T cells through multiple extracellular desertification and hijack a physiologic mechanism of immune self-tolerance.

Meanwhile, Tregs also utilized glycolysis to fuel additional rapid energy demand by enhancing mTOR activity with outlier stimulation. The Tregs F-actin polarization and formation of leading-edge synapses were enriched with phospho-AKT, thereby facilitating the directed migration upon chemokine stimulation^177^. CCR4 induced glucose uptake in Tregs was parallelly accompanied with PI3K/mTORC2 cascade induced downstream enzyme glucokinase (GCK) activation, consequently enhancing glycolysis engaged for Tregs migration 178. Synchronously, GCK could interact with actin to enhance cytoskeletal reorganization during CKRs-stimulated Tregs migration^179^. Glycolytic enzymes including GCK interact with actin to act as a glycolytic ATP feeder in Tregs, thus generating energy required for cytoskeletal rearrangements and migratory force. Unlike other hexokinases, GCK has low affinity manifestation to glucose, which might avoid glycolysis metabolites perturbations and maintain glycolytic flux of Tregs in tumor milieu, giving Tregs an advantage in harsh tumor milieu 180. The co-localization of ATPase and GCK to the extracellular membrane of immune synapses was observed during actin elongation and morphological changes, whereas unstimulated Tregs displayed reduced F-actin and GCK expression^4^. In various cells, mTORC2 activation is positively linked with Myc expression, rather mTORC1, thereby regulating the glucose transport and glycolytic flux^181^. In terms of this, mTORC2 could regulate the glycolytic flux by fully activating AKT and controlling Myc and GCK expression, consequently modulating Tregs intracellular glycolysis rate-limiting steps 178. Notably, inhibition of obligatory mTORC2 component Rictor could decrease cell chemotaxis and cytoarchitectural reorganization in an actin blunting-dependent manner^182^, which in Tregs manifests as unpolarized cell morphology with limited size and poorly formed immune synapses^4^. Although single CKRs may not be sufficient for Tregs recruitment and function in tumors, specific CKRs activation and signaling transduction are required for Tregs cytoarchitectural reorganization and direct/indirect full immunosuppressive function in tumor^163^.

### Tumor associated macrophages (TAMs)

**Figure 5:**
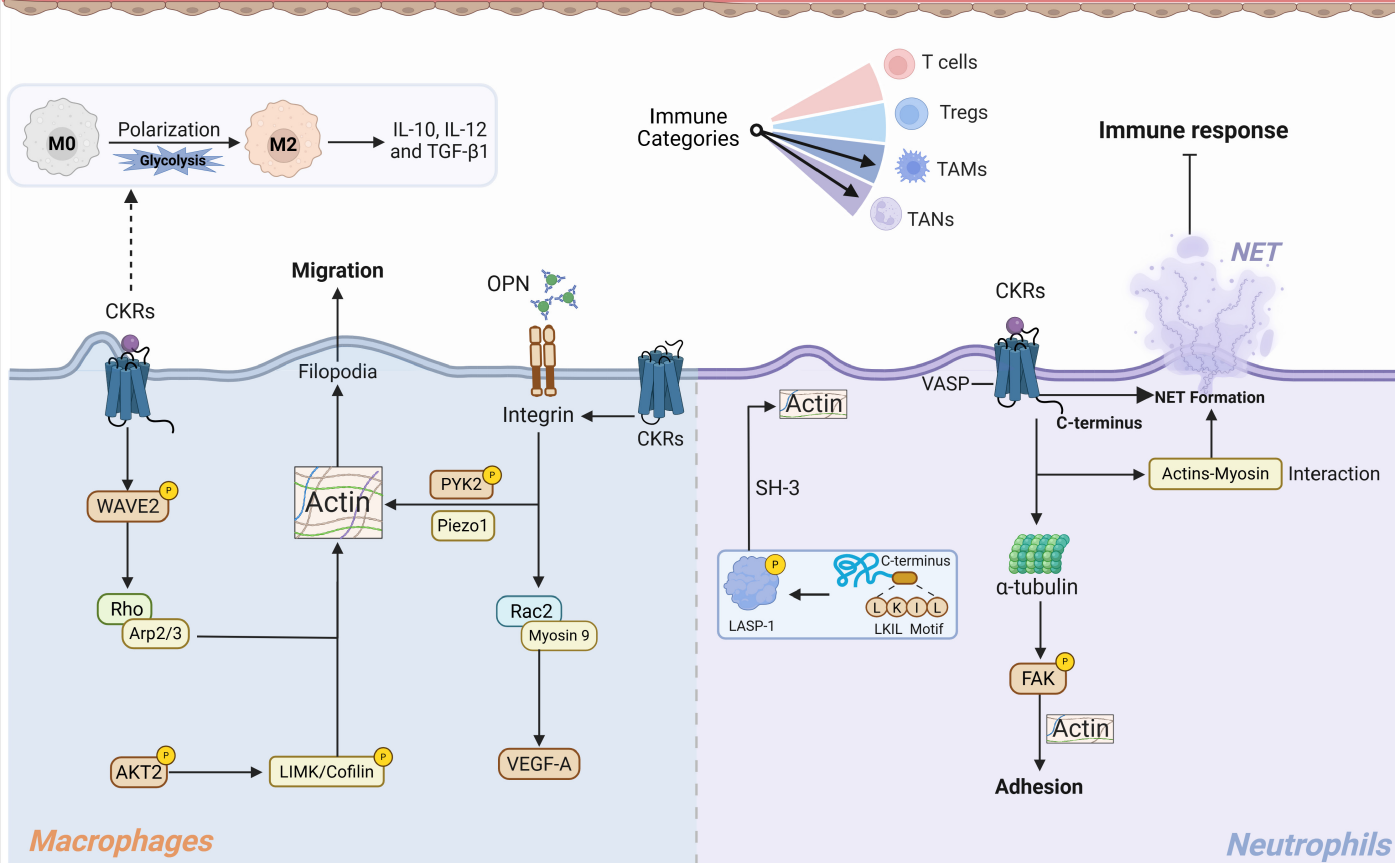
**Molecular explanation for CKRs signaling regulated actin networks in TAMs and TANs cells migration and functions**. CKRs activation promoting the TAMs and TANs migratory morphology and pro-tumor functions. **I.** TAMs polarization and migration: Activated CKRs promoting the PI3K/AKT2, Rac2, and WAVE2/Arp2/3 complexes signaling, consequently driving the actin nucleation and branched polymerization. Simultaneously, CKRs involved integrin signaling could enhance interaction between mechanosensor Piezo1 and PYK2 to regulate actin polarization and actomyosin formation. CKRs induced glycolysis-dependent M2-like TAMs polarization and the immunosuppressive cytokines secretion. **II.** TANs chemotactic morphology and migration: Chemokine-CKRs recognition could promote C-terminus LKIL motifs activation and LASP-1 phosphorylation to drive TANs actin polymerization. After CKRs activation, the VASP, α-tubulin, and local FAK signaling were synergistically to modulate TANs adhesion-contraction actin network and provide the adhesive migration force. Meanwhile, actin-myosin interaction driving the formation and of NETs and immunosuppressive function within TME. TAMs: Tumor associated macrophages, TANs: Tumor-associated neutrophil, NETs: Neutrophil extracellular traps, OPN: Osteopontin, VASP: Vasodilator-stimulated phosphoprotein, LASP-1: LIM and SH3 protein 1.

Macrophages are types of immune cells in defending against invading pathogens, and they participate in organ development, remodeling, healing and disease (**Figure 5**). Macrophages are also found in tumors with deleterious effects. In the tumor progression, macrophages density is positively correlated with microvessel counts and negatively correlated with patient prognosis^183^. Actin-rich structures in macrophages, such as basal podosomes and apical synapses, are essential for actin-dependent chemotaxis, phagocytosis, and cell-cell interactions within tumor foci^184^. Huang *et al*. observed that M2-like TAMs exhibit a tighter podosomes organization with compact actin polymerization fibers than M1 type^185^. Beyond directing macrophage migration, chemokine-CKR signaling drives macrophage polarization toward an M2-like TAM phenotype^186^, which is potentially associated with actin reorganization, cell elongation and spindle-shaped morphology functions^187^. In the mouse model, CXCR2-dominated macrophages accumulation was markedly rescued under the CXCL1-inhibition, accompanying with selectively upregulated aerobic glycolysis and tumor adaptability^188^. In macrophages, glycolytic ATP synthesis localizes in filopodia and lammelipodia, where ATP is rapidly consumed during actin remodeling process and continuously membrane protrusions formation during the adhesion movement^99^, ^105^. Yuan *et al*. indicated that CCL5 skewed M0 macrophages toward an M2-like TAMs phenotype and migration, as well as promoted macrophages IL-10, IL-12 and TGF-β1 in tumor 189. Of note, interleukin stimulation triggers the reorganization of actin-rich podosomes into rosette structures within M2 macrophages, enabling degradation of-and migration force through-dense extracellular matrices 190. Similarly, CCL2 and CXCL12-induced M2-like TAMs polarization and recruitment also were observed in colorectal cancer^191^. There has been well documented for TAMs that could play a pro/M2- or an anti/M1-tumoral function. The CCL5/CCL2-producing tumor cells were able to chemoattract TAMs that co-expressed M1/M2 markers^192^, ^193^. For the intracellular structure, osteopontin (OPN) and actin expression levels are correlated with TAMs infiltration, wherein the OPN acts as the potent chemokine^194^. The integrin is highly expressed on TAMs and constitutes a major OPN receptor and M2 macrophages took on a more adhesive phenotype than M1-type^194^, ^195^. Chemokines exerted integrin activation has been approved in various cell types, including immune and tumor cells^196^. The CCR2 and integrin engagement leads to an interaction between activated Rac2 and Myosin 9, and promoting macrophages vascular endothelial growth factor A (VEGF-A) expression^197^. Meanwhile, Myosin 9 accumulated in the leading-edge lamellipodia extensions of macrophages (generated by Rac-induced actin polymerization) in a manner dependent on its motor activity, contributing to an elongated and enlarged morphology 198. The Myosin 9 deficiency significantly weakened the CCL2-coupling in macrophage. Moreover, chemokine CCL18 stimulated PYK2 phosphorylation in TAMs potentially enhanced TAMs trans-endothelial migration and cancer-promoting functions^199^. The lack of PYK2 directly impairs the macrophages lamellipodia contractile activity and the forward immobilizing force during adhesion migration 200.

As a mechanical checkpoint, endogenous PYK2 could interact with stimulated CKRs to coordinate ordered F-actin polarization, which drives cellular force generation and significantly maintains TAMs stiffness and population^201^. The PYK2 senses mechanical signals via Piezo1 and integrins, triggering F-actin polymerization and translocating to the nucleus to regulate mechano-transduction and differentiation genes, thereby promoting monocyte-to-TAMs differentiation and recruitment^201^.

The Arp2/3 complex is required for macrophage integrin functions and chemotaxis, forming networks involved in lamellipodia protrusion, phagocytosis, and cell adhesion^202^. In addition to an enlarged and elongated morphology after macrophage chemotactic activation, the Arp2/3-branched actin polymerization at leading edge protrusion facilitated the processive advancement force. The loss of Arp2/3 activation suppressed the actin nucleation and lamellipodia in favor of filopodia, thereby decreasing spread cell area and altering macrophages motility shape^202^. Recent experiments underscore the fundamental role of F-actin, not only in mediating protrusion but also in spatially organizing signaling at the phagocytic cup. The altered lamellipodia protrusion/retraction cycles and defects in migration persistence that deregulate the real chemotaxis drivers, a process wherein lamellipodia generates cytoskeletal struts that push against the membrane to drive leading edge advancement and boost cell speed^203^. The WAVE2 directly activated Arp2/3 has been observed in immune cells chemokine coreceptor CXCR4 or CCR5 dependent signaling, and leads to actin nucleation and filament branching^204^. The Rho–formin and Rho family–WASP–ARP2/3 pathways are involved in formation of filopodia and invadopodia (short actin-filled protrusions that degrade extracellular matrix)^204^. This WAVE2 phosphorylation event was involved in both CKRs stimulated Gαi-dependent and -independent pathways^204^. On the other hand, TAMs activation/polarization to distinct functional states is critically supported by metabolic shifts. The CKRs like CCR5 activation stimulated PI3K/AKT has been demonstrated to promote M2 macrophage polarization, thereby promoting tumor progression 205. Upon activation of TAMs surface CKRs, a spectrum of phosphorylation cascades induces formation of actin filaments in the front and myosin contractile rings at the back and enhances cell adhesion and forward force^4^, ^98^, ^99^, ^102^. AKT was important in orchestrated actin cytoskeleton reorganization during cell migration, wherein AKT2 disruption will inhibit the downstream LIMK/Cofilin phosphorylation and directly contribute to the defects in TAMs actin polymerization and chemotaxis 206. In summary, TAMs chemotaxis is a spatiotemporally orchestrated response in which a spectrum of phosphorylation cascades couples metabolic shifts with targeted actin reorganization, mechanisms essential for sustaining front-rear polarity and driving forward adhesive movement.

### Tumor-associated neutrophil (TANs)

Neutrophils are the first immune cells to be recruited in response to infection or tissue injury to protect the host from harmful agents 207. Chemotaxis-stimulated neutrophils presented with F-actin polymerization at the leading-edge synapsis and integrin conformational change during the adhesion migration^208^. Meanwhile, growing evidence suggests a crucial regulatory role for neutrophils in tumor establishment and progression^209^. Tumor-associated neutrophils (TANs) exhibit morphological and functional heterogeneity, their plasticity mediating bidirectional responses depending on the surrounding TME 210. CXCR2 modulated tumor-associated neutrophil (TANs) infiltration has been demonstrated to be negatively associated with tumor prognosis^211^. The CXCR2 signaling was directly involved in F-actin and α-tubulin levels, as well as phagocytic ability 212. Of note, ligand CXCL1 induced neutrophil adhesion and transmigration were highly relay on β2 integrin-dependent adhesion and actin polymerization, thereby forming larger size and more extracellular synapses^213^. Similarly, reduced extracellular CXCL8 decreases both β2 integrin levels and adhesion strength in TANs to the same extent, thereby reducing the anchoring of entrapped cells^214^. Chemokine stimulation-induced cytoskeletal reorganization promoted neutrophil spreading, wherein F-actin-rich lamellipodia are found at the leading edge 215. Álvaro *et al*. reported that CXCR1/2 stimulation in TANs promoted neutrophil extracellular traps (NETs) formation, thereby inhibiting the immune response^216^. F-actin dynamics and myosin II function are essential for stimulus-induced NET formation, accompanying characteristic cell rounding and apparent contraction 215. This may significantly contribute to the intriguing morphological phenomenon where TANs transition from a homogeneous to a more plastic and heterogeneous state following the chemotactic attraction by tumor cells. The CXCR2 signaling activation is essential for neutrophils mature and structural proteins integrity, and the lack of CXCR2 stimulation will contribute to F-actin polymerization and α-tubulin decrease^217^. The actin-tubulin cytoskeleton reorganization in neutrophils is essential for chemotaxis-induced phagocytosis and maturity 212. CXCR2 expression by TANs is required for their homing to tumors where CXCR2 ligands are overexpressed 218. During inflammation, CXCL8 inhibition was proved to abolish actin polymerization, thereby eliminating CXCR2 dependent neutrophils chemotaxis^219^. The CXCL8-induced cytoplasmic calcium concentration upregulation, local intracellular actin high-density polarization and cell rounding were important in neutrophils recruitment 219. Meanwhile, N-terminal region of CXCR2 to CXCL8 epitope interaction was important for TANs chemotaxis and neutrophil extracellular trap (NET) formation^220^, ^221^. After stimulation, actins-myosin interactions, myosin that concentrated together with cortical actin along the periphery of the cell body forming the force to support NET expulsion^215^, ^222^.

Furthermore, in tumor adaptation, vasodilator-stimulated phosphoprotein (VASP) has been reported as a promising biomarker 223. As a processive actin polymerase, it promotes the formation of invasive membrane structures, leading to extracellular matrix remodeling. Neel *et al*. reported that VASP interacted with CXCR2 after CXCL8 stimulation in neutrophils, wherein free actin barbed ends will recruit VASP to the leading edge and phosphoprotein^224^. Similarly, in CXCR1- and CXCR2-expressing neutrophils, actin and microtubules were required for CXCL8-induced focal adhesion kinase (FAK) phosphorylation^225^. The phosphorylation of FAK was adhesion-dependent and was highly reliant on actin filaments and cytoskeleton reorganization to provide contractility at cell-substratum contact regions. Moreover, LIM and SH3 protein 1 (LASP-1), binds CXCR2 under both basal and ligand activated conditions, and co-localize at the leading edge of migrating cells and mediates cell chemotaxis 226. The CXCR2 intracellular C-terminus LKIL motif (aa 327–330) interacts with the LASP1 and promotes LASP1 phosphorylation 226. LASP-1 is an actin binding cytoskeletal protein, which potentially increases the neutrophils actin protein levels by inhibiting the depolymerization of actin^227^, ^228^. The LASP-1 contributes to cellular immunity and adhesion mechanisms, and predominantly binding to actin occurs via SH3 domain and the first nebulin repeat, and localized at the plasma membrane and in actin-rich subcellular protrusive structures 229. These intricate coupling of chemokine signaling with dynamic cytoskeletal underlies remodeling the morphological plasticity of TANs, ultimately shaping their complex immune activities and adaptive roles within the TME.

## Therapeutic implications and clinical translation

The developing landscape of tumor immunology highlights that immune cell chemotaxis is governed by an intricate "biomechanical-metabolic" axis, revealing novel mechano-checkpoint for clinical intervention. Recent studies reveal that a mechanosensitive immune checkpoint regulated by CKR signaling plays a direct role in immune-synapse cytoarchitecture and the efficacy of tumor immunotherapies^35^, ^37^, ^95^. Through distinguish the CKRs-dependent actin polymerization and integrin adhesion signaling may achieve the dual-pronged strategy that synchronously enhancing the effector CD8^+^ T cells physical penetrance and paralyzing immunosuppressive populations^5^, ^199^, ^201^, ^202^. Furthermore, due to actin-based chemotaxis is bioenergetically demanding, decoupling the metabolic-cytoskeletal connection presents another critical therapeutic strategy. Targeted intervention in either the mTORC2/GCK-mediated glycolysis essential for Treg immune synapses and migratory morphology^4^, ^178^, or the PFKFB3-driven glycolytic flux required for TAM podosome formation and intercellular contact forces^102^, ^103^, can selectively inhibit immune cell chemotaxis. Concurrently, alleviating TME deprivation or targeting metabolic supplementation can rescue the morphological fitness of exhausted CD8^+^ T cells^114^, ^115^, re-establishing their structural capacity to execute cytotoxic functions.

CKR-directed biomechanical-metabolic therapies have great potential for translation into clinical practice. Advanced delivery systems, such as tumor-targeted magnetic nanomotors^33^ or stimuli-responsive polymeric nanoparticles, offer a sophisticated solution for locally disrupting intracellular actin networks or purinergic signaling in immune cells within the TME^81^, ^82^. These nanotechnology-based interventions can effectively improve immune cells mechanical microenvironment and alter cytoskeletal affinities without broad off-target effects, which overcoming systemic toxicity and the redundancy of chemokine networks. Crucially, these mechano-modulators are highly compatible with combinatorial regimens. Pharmacologically activating specific CKRs and biomechanical signaling promotes initial effector T cell homing, which transforms immunologically 'cold' tumors into 'hot' ones and establishes a structural prerequisite that might synergistically amplifies the efficacy of anti-PD-1/PD-L1 therapy^5^, ^90^. To develop precision immunotherapy strategies, future research should focus on high-resolution dynamic imaging technologies and elucidate chemotaxis-induced cytoskeletal biomechanical regulation mechanisms, thereby strategically rewiring the chemotaxis, biomechanical, and metabolic forces that drive immune cells trafficking.

## Conclusions

As evidence has been shown from the literature, chemokines are extensively studied and greatly contribute to the tumor immune reshapes. With the growing understanding of the biology and biogenesis of CKRs signaling in immune cells migration and actin cytoskeleton alteration, chemotaxis research has generated much excitement in the past decade. An important task is to link findings obtained with different experimental cues, taking into account CKRs conformations, signal transduction and cytoskeleton molecules parameters, as well as post-activation metabolic and polarization of TME immune cells, to reveal immune cells chemotaxis specific and activation signaling networks. Chemokines trigger receptor to activate context-specific signaling pathways through selectin, integrins, Ca^2+^, and actin modulation, and that in immune cells these functionally different cytoskeleton-binding proteins work in synergy with each other to promote morphology dynamics and force. In particularly, CKRs stimulation-induced immune cells migration appears to require dynamic interplay between actin polymerization and flux membrane protrusion, while metabolite from CKRs-induced metabolic reprogramming substantially contributes to the concentration of leading front actin filaments and flow-forced traction forces in cells. However, the underlying molecular mechanisms are still incompletely understood as the lacking of dynamic-resolution structures and signaling transduction in the stimulation of CKRs. How functionally distinct cytoskeleton molecules structures and cortex alteration of tumor immune cells response to CKRs signaling are only beginning to understand, as exemplified by the coordinate regulation of lamellipodia actin/integrins network and cell migration tractive force.

With critical roles in immune cells structural biochemical regulation, migration and immunosuppressive TME formation, the chemokine system embodies a key therapeutic target for improving tumor immune disorder. Here, we outlined the potential CKRs signaling cascades for immune cells migration that interfere with intracellular function through structural biomechanics and metabolic reprogramming signals. By modulating actin cytoskeleton reorganization, downstream integrin clustering, recruitment of metabolic reprograming for the morphological adaption, the CKRs help potentiate signaling in immune cells, thereby serving as trigger and modulators of immune cells migration required for functional responses in TME. Immune cells trafficking and effector functions within the TME are highly regulated process involving unique and complex combinations of chemokine stimulation, cellular mechanical force and intracellular cytoskeleton networks. Thus, we anticipate that future studies using advanced imaging methods, combined with biophysical approaches, will reveal interesting new insights into mechanical regulation of the chemokine-derived immune cells migration in the TME context and will uncover new links between biochemical and mechanical regulation of the cytoskeleton-reorganization. While efforts are still required to optimize these gaps, this anticipates great potential for the design of future tumor immunotherapy aimed at the chemokine system.
